# Repeated porphyrin lipoprotein-based photodynamic therapy controls distant disease in mouse mesothelioma via the abscopal effect

**DOI:** 10.1515/nanoph-2021-0241

**Published:** 2021-08-03

**Authors:** Jenny Lou, Masato Aragaki, Nicholas Bernards, Tomonari Kinoshita, Jessica Mo, Yamoto Motooka, Tsukasa Ishiwata, Alexander Gregor, Tess Chee, Zhenchian Chen, Juan Chen, Kichizo Kaga, Satoru Wakasa, Gang Zheng, Kazuhiro Yasufuku

**Affiliations:** Department of Medical Biophysics, University of Toronto, PMCRT 5-354, 101 College Street, Toronto, Ontario M5G 1L7, Canada; Princess Margaret Cancer Centre, University Health Network, Toronto, Ontario M5G 1L7, Canada; Division of Thoracic Surgery, Toronto General Hospital, University Health Network, 200 Elizabeth Street, EN 9N‐957, Toronto, Ontario M5G 2C4, Canada; Department of Cardiovascular and Thoracic Surgery, Hokkaido University Faculty and School of Medicine, Sapporo, Hokkaido 060-8638, Japan; Division of Thoracic Surgery, Tachikawa Hospital, 4-2-22 Nishikicho, Tachikawa, Tokyo, 190-8531, Japan; Department of Pharmacology and Toxicology, University of Toronto, Toronto, Ontario M5S 1A8 Canada; Department of Thoracic Surgery, Kumamoto University Hospital, 1-1-1 Honjo, Chuo-ku, Kumamoto, 860-8556, Japan; Faculty of Health Sciences, McMaster University, Hamilton, Ontario L8S 4L8, Canada

**Keywords:** immune response, immunotherapy, PD-1, photodynamic therapy, porphyrin, thoracic malignant tumor

## Abstract

While photodynamic therapy (PDT) can induce acute inflammation in the irradiated tumor site, a sustained systemic, adaptive immune response is desirable, as it may control the growth of nonirradiated distant disease. Previously, we developed porphyrin lipoprotein (PLP), a ∼20 nm nanoparticle photosensitizer, and observed that it not only efficiently eradicated irradiated primary VX2 buccal carcinomas in rabbits, but also induced regression of nonirradiated metastases in a draining lymph node. We hypothesized that PLP-mediated PDT can induce an abscopal effect and we sought to investigate the immune mechanism underlying such a response in a highly aggressive, dual subcutaneous AE17-OVA+ mesothelioma model in C57BL/6 mice. Four cycles of PLP-mediated PDT was sufficient to delay the growth of a distal, nonirradiated tumor four-fold relative to controls. Serum cytokine analysis revealed high interleukin-6 levels, showing a 30-fold increase relative to phosphate-buffered solution (PBS) treated mice. Flow cytometry revealed an increase in CD4+ T cells and effector memory CD8+ T cells in non-irradiated tumors. Notably, PDT in combination with PD-1 antibody therapy prolonged survival compared to monotherapy and PBS. PLP-mediated PDT shows promise in generating a systemic immune response that can complement other treatments, improving prognoses for patients with metastatic cancers.

## 1 Introduction

Photodynamic therapy (PDT) is a minimally invasive, clinically approved cancer treatment for melanoma, non-small cell lung, head and neck, and esophageal cancers. Intravenously administered photosensitizers accumulate at the target tumor, which can then be irradiated with a photosensitizer-specific wavelength of light to activate the photosensitizer [1, 2]. Subsequent generation of reactive molecular species can induce different mechanisms of tumor cell death, including necrosis, apoptosis, autophagy, and paraptosis, depending on photosensitizer type and subcellular localization [3, 4]. Damage-associated molecular patterns are secreted by dying cells, as are cytokines like interleukin-1 beta (IL-1β), interleukin-6, and tumor necrosis factor-alpha (TNF-α). This process, termed immunogenic cell death (ICD), is thought to be an initiating step in generating an adaptive immune response that can induce systemic antitumor immunity.

Immediately after PDT, neutrophils infiltrate the irradiated tumor [5], [6], [7]. Neutrophils migrate to tumor-draining lymph nodes, where they interact with dendritic cells and T cells [8]. A key immune cell responder is the dendritic cell, which processes tumor-associated antigens at the tumor site [4]. Next, dendritic cells migrate to the draining lymph nodes and educate naive T cells to recognize specific antigens. Primed CD4+ T cells can provide help to CD8+ T cells, which migrate to the tumor and attack tumor cells that express specific antigens. A subpopulation of these primed CD4+ and CD8+ T cells differentiate into central and effector memory T cells. Upon a secondary encounter with the target antigen, these memory T cells can utilize various effector functions to attack tumor cells.

Due to the different mechanisms of cell death induced by PDT, it holds potential for use in combination with immunotherapies. PDT with various photosensitizers have been explored in combination with immune checkpoint inhibitors, such as αCTLA-4 or αPD-1 antibodies [9], [10], [11]. PDT may synergize with immunotherapies to bolster antitumor efficacy at primary irradiated tumors and promote systemic immune responses against metastases [12, 13]. Ideally, immune memory would be generated to prevent relapses and help patients achieve long term remission.

Previously, we developed the porphyrin lipoprotein (PLP) platform: A stable, ∼20 nm biomimetic nanoparticle with a hydrophobic core amenable to drug loading, and a porphyrin-lipid monolayer shell [14]. This multimodal nanoparticle can integrate positron emission tomography, fluorescence imaging, and PDT into one platform. Notably, we observed that following PLP-mediated PDT in a metastatic VX2 buccal carcinoma rabbit model, eradication of the irradiated VX2 tumor also coincided with regression of a lymph node metastasis, despite lack of laser irradiation at this site [15]. This observation raised the feasibility of PLP-mediated PDT to generate an abscopal effect, in which treatment of one tumor generates a systemic immune response that can control or eliminate a secondary, untreated tumor.

Here, we aim to: (1) determine the circumstances in which PLP-mediated PDT can effectively induce immune responses, (2) investigate the immune mechanism underlying such an immune response, and (3) evaluate whether combination therapy can further improve survival outcomes. We determined that four cycles of PLP-mediated PDT delayed growth of a distal, nonirradiated tumor in a highly aggressive dual-subcutaneous AE17-OVA+ mesothelioma model. This tumor growth delay was underpinned by elevated serum interleukin-6 (IL-6) levels, a lower percentage of central memory CD4+ T cells in the spleen, and a larger percentage of CD4+ T cells and effector memory CD8+ T cells in nonirradiated tumors. Lastly, combination PDT with αPD-1 antibody therapy mediated prolonged survival relative to monotherapy and PBS. PLP-mediated PDT holds potential to generate an immune response that can be harnessed by combination therapy, to produce superior outcomes for patients with metastatic tumors.

## 2 Methods

### 2.1 Materials

Lipids, 1,2-dimyristoyl-sn-glycero-3-phosphocholine (DMPC) and cholesteryl oleate, were purchased from Avanti Polar Lipids Inc. Cell culture media, Roswell Park Media 1640 (RPMI-1640) and Dulbecco’s Modified Eagle Medium (DMEM) were obtained from Gibco. Supplemental fetal bovine serum (FBS) and penicillin/streptomycin was purchased from Gibco. The following flow cytometry antibodies were purchased from BioLegend: PE antimouse CD3ε antibody, PerCP/Cyanine5.5 antimouse CD4 antibody, APC/Cy7 antimouse CD8a antibody, and BV 510 antimouse CD62L antibody. BV 605 antimouse CD44 antibody was purchased from Thermo Fisher. DAPI and TruStain FcX™ (antimouse CD16/32) antibody were purchased from BioLegend. Collagenase IV and DNAse I were obtained from Sigma Aldrich and Thermo Scientific, respectively. α-PD-1 antibodies for combination therapy were purchased from BioXCell (clone RMP-14). An anti-calreticulin antibody was purchased from Novus Biologicals (Calreticulin Antibody, NB600-103).

### 2.2 PLP synthesis

PLP was synthesized, according to the protocol described by Cui et al. [14]. Briefly, a lipid film consisting of 0.9 µmol porphyrin-lipid, 2.1 µmol DMPC, and 0.3 µmol cholesteryl oleate was formed. The film was subsequently hydrated with PBS (150 mM, pH 7.5) and bath sonicated for 1 h. R4F peptide (2.3 mg; 5 mg/mL) was added dropwise to the solution, after which the turbid solution became transparent. The next day, the solution was centrifuged at 12 000 rpm for 20 min, and the supernatant was filtered through a 0.1 µm membrane (Millex^®^, Sigma-Aldrich).

### 2.3 PLP characterization

The morphology and size of PLP was assessed using transmission electron microscopy (TEM). Samples were diluted 50× in ddH_2_O, placed on grids (Formvar/Carbon Square 400 Mesh, Ultra-Thin ‘B’; Electron Microscopy Services), washed with ddH_2_O, and stained with 1% uranyl acetate for 30 s, and imaged under an electron microscope (T20; FEI Tecnai). To assess the absorbance of PLP, ultraviolet–visible spectroscopy was conducted (CARY). Intact samples were diluted 400× in PBS and disrupted samples were disrupted in methanol and diluted 400×. Absorbance spectra were collected from 200 to 800 nm. Fluorescence quenching efficiency was used as a proxy for the stability of PLP. Samples were diluted in PBS or FBS with or without 1% TritonX-100 to a final concentration of 25 μM and scanned over 24 h (CLARIOstar). A final volume of 50% FBS was used to mimic *in vivo* serum conditions. PLP samples were excited at 410 nm, and emission was collected from 500 to 799 nm (*n* = 3). To assess the fluorescence spectra of PLP, samples diluted in PBS or disrupted in 1% TritonX-100 were excited at 410 nm and collected from 500 to 799 nm (*n* = 3).

### 2.4 Cell culture

Human A549 adenocarcinoma cells and H2170 squamous carcinoma cells were kindly gifted by Dr. Ming-Sound Tsao (University of Toronto, Toronto, Ontario, Canada). A549 cells were cultured in DMEM supplemented with FBS (10% v/v), while H2170 cells were cultured in RPMI-1640 media supplemented with FBS (10% v/v), 100 U/mL penicillin, 100 mg/mL streptomycin and nonessential amino acids. AE17-OVA+ mesothelioma cells were kindly provided by Dr. Marc de Perrot (University of Toronto, Toronto, Ontario, Canada). AE17-OVA+ cells were cultured in RPMI-1640 media, supplemented with FBS (5 v/v%), 100 U/mL penicillin, 100 mg/mL streptomycin and nonessential amino acids. All cells were cultured at 37 °C in 5% CO_2_.

### 2.5 *In vivo* testing

All animal experiments were approved by the University Health Network Animal Care Committee and were conducted in adherence with all relevant institutional, provincial, and federal requirements.

#### 2.5.1 Tumor models

A549 and H2170 tumor models: Female athymic nude mice (6–10 weeks old, 17–22 g weight) were anesthetized with isoflurane (5% induction; 2.5% maintenance). 1 × 10^6^ A549 or H2170 cells were resuspended in PBS and aspirated into a 25-gauge needle. Cells were injected subcutaneously into the dorsal, left hindlimb of mice. Subsequent tumor growth was monitored with calipers. Tumor volumes were calculated as:

$$\text{tumor volume}=\text{length}\times {\text{width}}^{2}/2$$

AE17-OVA+ model: In preparation for tumor inoculations, immunocompetent female C57BL/6 mice were anesthetized with isoflurane, and the hair on the hindlimbs were shaved (Peanut shaver), and subsequent application of hair removal cream (Nair). The next day, mice were anesthetized using isoflurane (5% induction, 2.5% maintenance). With a 25-gauge needle, mice were inoculated with 1 × 10^6^ AE17-OVA+ mouse mesothelioma tumor cells resuspended in PBS, into both dorsal hindlimbs. Tumor growth was monitored with calipers. Tumor volumes were calculated as described above.

#### 2.5.2 PLP biodistribution

The biodistribution of PLP was assessed by fluorescence imaging in a dual subcutaneous AE17-OVA+ tumor model. Three days prior to imaging, mice were fed low-fluorescence diet (Harlan–Tekland). At 48 and 24 h prior to imaging, mice were intravenously injected with PLP (4 mg/kg porphyrin-lipid) in the tail vein. At 24 and 48 h postinjection, mice were anesthetized with isoflurane (5% induction; 2.5% maintenance). Whole body fluorescence imaging was performed on the CRi Maestro imaging system (Caliper Life Sciences). An excitation filter of 616–661 nm was used, while a 675 nm longpass emission filter was employed. Subsequently, mice were euthanized with 5% isoflurane and cervical dislocation. Organs including the liver, spleen, muscle, right and left hindlimb tumors, heart, lungs, and kidneys, were removed and underwent *ex vivo* fluorescence imaging with the same filter set.

#### 2.5.3 Photodynamic therapy

In A549 and H2170 tumor bearing mice, PLP (4 mg/kg) was intravenously injected 24 h prior to photodynamic therapy. Immediately before laser irradiation, mice were anesthetized with isoflurane and positioned on the base of a custom laser set-up. An image of the set-up is provided in Supplementary Figure 2. A 671 nm laser was used to irradiate mice. For the AE17-OVA+ dual tumor bearing mice, only tumors on the dorsal, left flank were irradiated. For optimization of light dosages, mice were irradiated at either 25, 50, or 75 J/cm^2^ (671 nm, 100 mW/cm^2^). For repeated cycles of PDT, mice were intravenously injected with PLP and irradiated either once, twice, three, or four times, as per treatment group. PLP injections were administered on days 0, 3, 7, or 10. The left tumors of mice were irradiated on days 1, 4, 8, and 11. Mice were sacrificed at their humane endpoint, which was defined as either loss of >20% of body weight, or the sum of both tumor volumes exceeding 1500 cm^2^.

#### 2.5.4 Combination therapy

The efficacy of combination PDT and αPD-1 antibody therapy were investigated in AE17-OVA+ dual tumor-bearing mice. Three days after tumor cell implantation, mice were randomly assigned into different treatment groups (PBS, PLP, αPD-1 antibody, and PLP +αPD-1 antibody, *n* = 6 per each group). PLP was injected intravenously and αPD-1 antibodies (12.5 mg/kg) were injected intraperitoneally on the same day. We defined the day of the first PLP and αPD-1 antibody injection as “day 0”. PLP and αPD-1 were injected four time in total (days 0, 3, 7, and 10), followed by laser irradiation (days 1, 4, 8, and 11). All mice received light irradiation (671 nm wavelength, 100 mW/cm^2^) for 5 min, at 24 h post injection of PLP and αPD-1 antibody. Tumor volumes were measured with calipers and mice were weighed. Mice were sacrificed at their humane endpoint, which was defined as either loss of >20% of body weight, or the sum of both tumor volumes exceeding 1500 cm^2^.

#### 2.5.5 Flow cytometry

AE17-OVA+ dual tumor bearing mice were randomly assigned into four different treatment groups (PBS, PLP, PBS + laser irradiation, or PLP + laser irradiation). Mice were intravenously injected with either PBS or PLP on days 0, 3, 7, and 10, and irradiated according to their treatment group 24 h after injection. On day 12, mice were anesthetized with isoflurane and sacrificed. Tumors and the spleen were removed from each mouse for flow cytometry.

To process the spleen into a single-cell suspension, mechanical digestion was employed. After filtration through a 100 μm cell strainer (Falcon), spleen samples were resuspended in erythrolysis buffer for 10 min. Following red blood cell lysis, samples were washed with PBS, centrifuged, and counted. Tumors were cut into small pieces and digested in collagenase IV and DNAse I. To stop the enzymatic reaction, EDTA was added. Subsequently, tumor cells were washed, centrifuged, and filtered.

Next, spleen and tumor cells were incubated with TruStain FcX™ (1:50 dilution) for 20 min at 4 °C to block Fc receptors. Next, samples were washed and centrifuged. Cells were stained with antibody panel consisting of PE CD3ε, PerCP/Cyanine5.5 CD4, APC/Cy7 CD8, BV 510 CD62L, and BV 605 CD44 for 20 min at 4 °C. After, samples were washed and centrifuged, and stained with a cell viability dye (DAPI). All samples were analyzed on a cytoFLEX S (Beckman Coulter, USA) and data were analyzed with FlowJo (TreeStar).

#### 2.5.6 Serum analysis

Mice were anesthetized and terminal cardiac puncture with a 25-gauge needle was performed. Approximately 500 µL of blood per mouse was obtained. Blood rested at room temperature for ∼45 min, to enable clotting. Then, samples were centrifuged at 1000 g for 10 min. The supernatant was collected and diluted in PBS two-fold. Samples were submitted to Eve Technologies (Calgary, Canada) for serum analysis via the mouse cytokine array proinflammatory focused 10-plex. Analysis of granulocyte-macrophage colony-stimulating factor (GM-CSF), interferon-gamma (IFNγ), interleukin-1 beta (IL-1B), interleukin-2 (IL-2), interleukin-4 (IL-4), interleukin-6 (IL-6), interleukin-10 (IL-10), interleukin-12p70 (IL-12p70), monocyte chemoattractant protein-1 (MCP-1), and TNF-α was performed. Meanwhile, serum globulins, alanine aminotransferase, amylase, total bilirubin, calcium phosphorus, and sodium were submitted to the Animal Resource Centre for scanning via the VetScan VS2 (Zoetis).

#### 2.5.7 Histology

After dissection, tumors, lymph nodes, and spleens were stored in 10% formalin for 3 days and subsequently transferred to 70% ethanol. Samples were submitted to the STTARR Histopathology Core services for paraffin embedding, slicing, and staining of hematoxylin and eosin, CD3, and calreticulin. Stained samples underwent whole slide scanning at 20× magnification (Aperio Scanscope XT whole-slide scanner, Leica Biosystems) at the Advanced Optical Microscopy Facility. Whole slide analysis was performed on stained samples using the cytonuclear analysis module on Halo (Indica Labs) to calculate the percentage of positively stained cells.

#### 2.5.8 Statistics

Statistical analysis was conducted with GraphPad Prism version eight software. Differences in tumor volumes between treatment groups were assessed with two-way repeated measures analysis of variance (ANOVA), followed by post-hoc Tukey’s multiple comparisons tests. Differences between treatment groups for survival curves were discerned with log-rank (Mantel–Cox) tests. For flow cytometry and histology data, ordinary one-way ANOVAs followed by post-hoc Tukey’s multiple comparisons test were used to discern differences in T-cell populations. Ordinary one-way ANOVAs and post-hoc Tukey’s multiple comparisons tests were also used to determine if differences between treatments were observed in serum cytokine levels. Significance was set at p < 0.05. Values are presented as mean ± standard deviation.

## 3 Results

### 3.1 PLP is an effective photosensitizer for photodynamic therapy in lung tumor models

**Figure 1:**
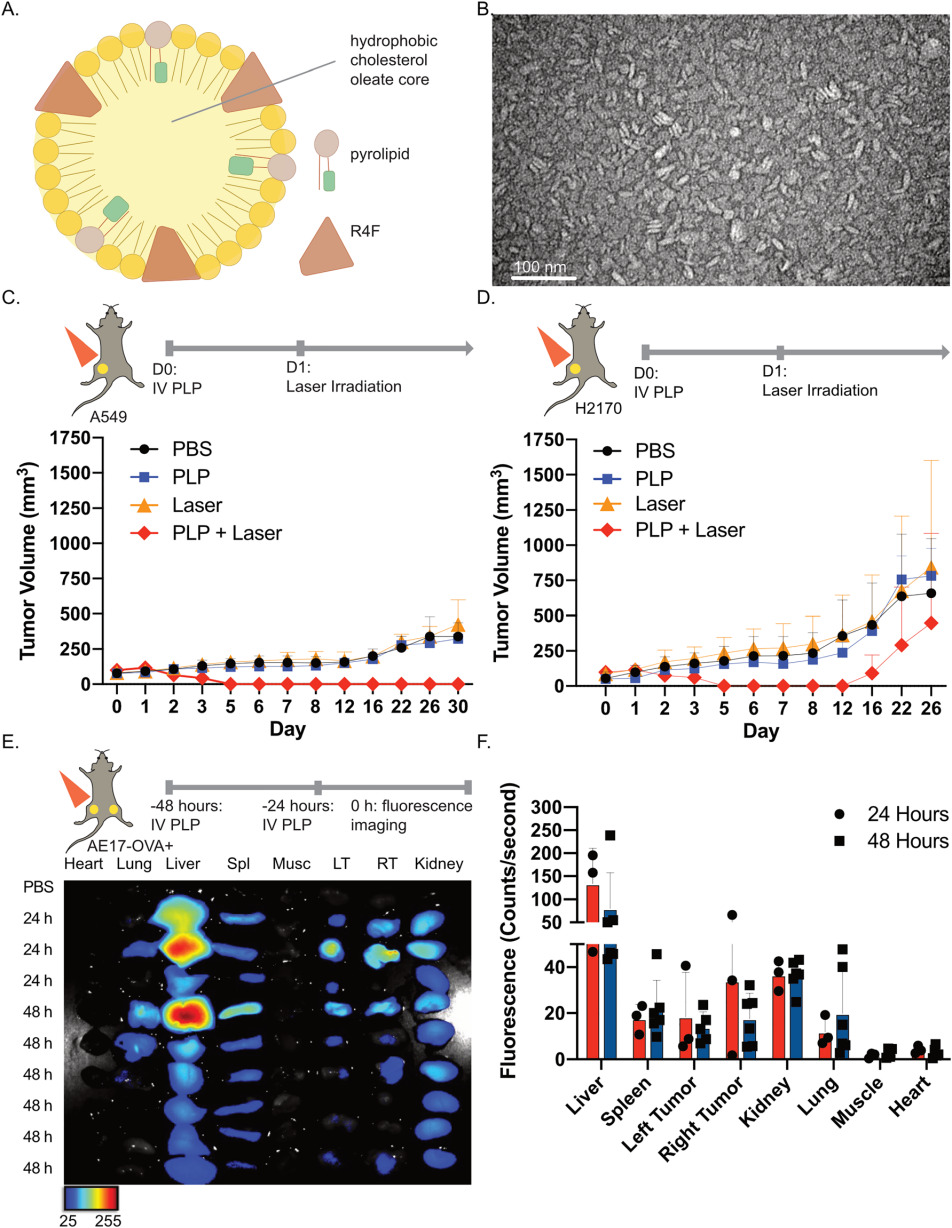
Porphyrin-lipoprotein (PLP) can be an effective photosensitizer for lung tumor models. (A) Schematic of PLP, the photosensitizer used for photodynamic therapy. (B) Transmission electron microscopy (TEM) image of PLP at 200 000× magnification. (C) Assessment of tumor burden in subcutaneous A549 tumors in nude mice, after treatment with phosphate-buffered solution (PBS), PLP (4 mg/kg), laser irradiation, or photodynmaic therapy (PDT) (PLP + laser). *n* = 3 per group. (D) Assessment of tumor burden in subcutaneous H2170 tumors in nude mice, after treatment with PBS, PLP (4 mg/kg), laser irradiation, or PDT (PLP + laser). *n* = 2 per group. Mice received light irradiation with a 671 nm laser at a light dose of 100 J/cm^2^ at 24 h postinjection of PLP. (E) Ex vivo fluorescence imaging of heart, lung, spleen (Spl), muscle (Musc), left (LT) and right (RT) tumors, and kidneys dissected from mice bearing dual subcutaneous AE17-OVA+ tumors. Mice received intravenous injections of PBS (*n* = 1), or PLP (4 mg/kg) at 24 h (*n* = 3) or 48 h (*n* = 6) prior to imaging. (F) Quantification of the fluorescence signal in various organs. Data are mean ± standard deviation.

PLP was formulated with porphyrin-lipid, R4F peptide, and cholesterol oleate (Figure 1A). Our previous study demonstrated that R4F enabled the formation of an α-helix peptide network that constrained both the size of PLP, and also stabilized the nanoparticle to generate favorable pharmacokinetics and biodistribution without the need for PEGylation. Nanoparticle morphology was characterized by transmission electron microscopy, which revealed that R4F successfully constrained the size of the nanoparticles to ∼20 nm in diameter (Figure 1B). PLP was further characterized (Supplementary Figure 1); nanoparticles demonstrated fluorescence quenching and remained stable in serum conditions after 24 h. Next, the efficacy of PLP-mediated PDT was evaluated in lung tumor models. In mice bearing subcutaneous A549 lung adenocarcinomas, a single round of PDT eradicated tumors (Figure 1C), whereas tumors continued to grow in mice treated with PBS, PLP, or laser irradiation alone. Similarly, in mice bearing H2170 lung squamous cell tumors, PDT initially eradicated tumors (Figure 1D). By 16 days post injection of PLP, the aggressive tumor line resulted in tumor regrowth, albeit at a delayed rate relative to control mice.

Given the efficacy of PLP-mediated PDT in these two lung tumor models, we next considered its application in a highly aggressive, immunocompetent AE17-OVA+ mesothelioma model. Mesotheliomas are highly aggressive tumors of the pleural and peritoneal cavities, often caused by asbestos exposure. Malignant pleural mesothelioma account for the majority of cases [16]. Treatment of malignant pleural mesothelioma centers on chemotherapy as most candidates are ineligible for surgical resection [17]. There is an unmet clinical need for improved treatment of malignant pleural mesothelioma, because the median survival of patients with surgically unresectable disease is ∼12 months after diagnosis [16].

To evaluate whether PLP has differential tumor accumulation in a dual subcutaneous AE17-OVA+ model in immunocompetent C57BL/6 mice, PLP was intravenously injected into mice at either 24 or 48 h prior to dissection and *ex vivo* fluorescence imaging (Figure 1E and F). The strongest porphyrin-lipid fluorescence signal was detected at 24 h post injection in the liver. As the site of PLP metabolism, the liver had the greatest signal. Fluorescence signal was also present in the tumor at 24 h, reflecting PLP accumulation. Importantly, porphyrin-lipid fluorescence was similar across both tumors. Fluorescence quantification did not detect a difference in bilateral tumor accumulation at either 24 or 48 h. Therefore, we proceeded with the 24 h timepoint for all subsequent PDT experiments.

### 3.2 Optimization of light dose to 50 J/cm^2^ to elicit an immune response

**Figure 2:**
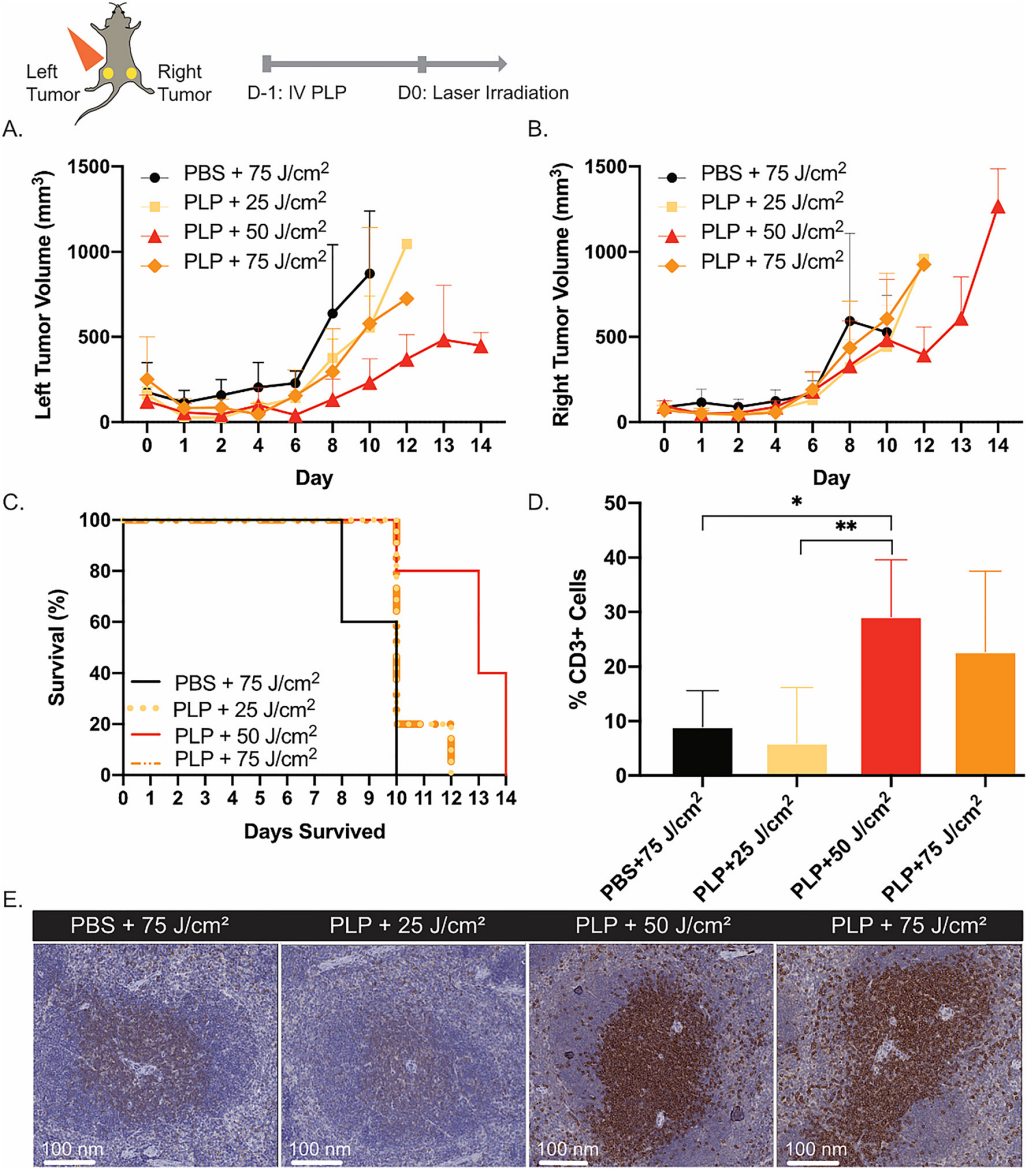
Optimization of light dosage for immune priming. (A) Dual subcutaneous AE17-OVA+ tumor bearing mice were treated with either PBS or PLP (4 mg/kg). Twenty-four hours post-injection, the left hindlimb tumors on mice were irradiated with a 671 nm laser at a light dose of either 25 J/cm^2^, 50 J/cm^2^, or 75 J/cm^2^. Evaluation of tumor volumes of irradiated left hindlimb tumor volumes (*n* = 5). (B) Assessment of non-irradiated right hindlimb tumor volumes (*n* = 5). (C) Survival of mice treated with either PBS + 75 J/cm^2^, PLP + 25 J/cm^2^, PLP + 50 J/cm^2^, or PLP + 75 J/cm^2^ (*n* = 5). (D) Histological analysis of the percentage of CD3+ T cells in spleens of mice harvested at time of endpoint (*n* = 3). (E) Microscopic image of a CD3-stained spleen from one mouse in each treatment group. Data are mean ± standard deviation. Statistical significance was determined using a one-way ANOVA followed by a post-hoc Tukey test. *p < 0.05, **p < 0.01.

High light dosages can damage tumor vasculature and reduce infiltration of innate immune cells, thereby limiting acute inflammation and subsequent antitumor immunity [18]. To determine the optimal light dosage for PLP-mediated PDT that can induce an immune response, dual subcutaneous AE17-OVA+ tumor bearing mice were injected with PLP (4 mg/kg), and the left tumors were irradiated 24 h later at either 25 J/cm^2^, 50 J/cm^2^, or 75 J/cm^2^. All mice that received PDT initially experienced tumor regression at the irradiated site, but the aggressive nature of AE17-OVA+ tumors subsequently prompted tumor re-growth (Figure 2A). Meanwhile, regardless of light dosage, the right tumors of mice that received PDT grew rapidly (Figure 2B). All mice reached their humane endpoint within two weeks of PDT. No difference in survival was observed between the groups (p > 0.05) (Figure 2C). Notably, histological analysis of CD3+ T cells in the spleen revealed that mice treated with PLP+ 50 J/cm^2^ had a 3.2-fold greater percentage of CD3+ T cells compared to the laser irradiated controls and 4.9-fold greater percentage of CD3+ T cells compared to mice treated with PLP + 25 J/cm^2^ (Figure 2D and E). Microscopy of CD3+ stained spleens revealed clustering of CD3+ T cells in the white pulp. After PDT at 50 J/cm^2^, there was greater CD3+ staining within the white pulp, which suggested that CD3+ T cells were expanding. For subsequent experiments, a light dosage of 50 J/cm^2^ was employed, because of its propensity for immune activation. The selection of 50 J/cm^2^ also aligned with an immune-enhancing protocol established by Shams et al. for HPPH-mediated PDT of 4T1 breast tumors [19].

### 3.3 Photodynamic therapy reduced calreticulin expression in AE17-OVA+ tumors

**Figure 3:**
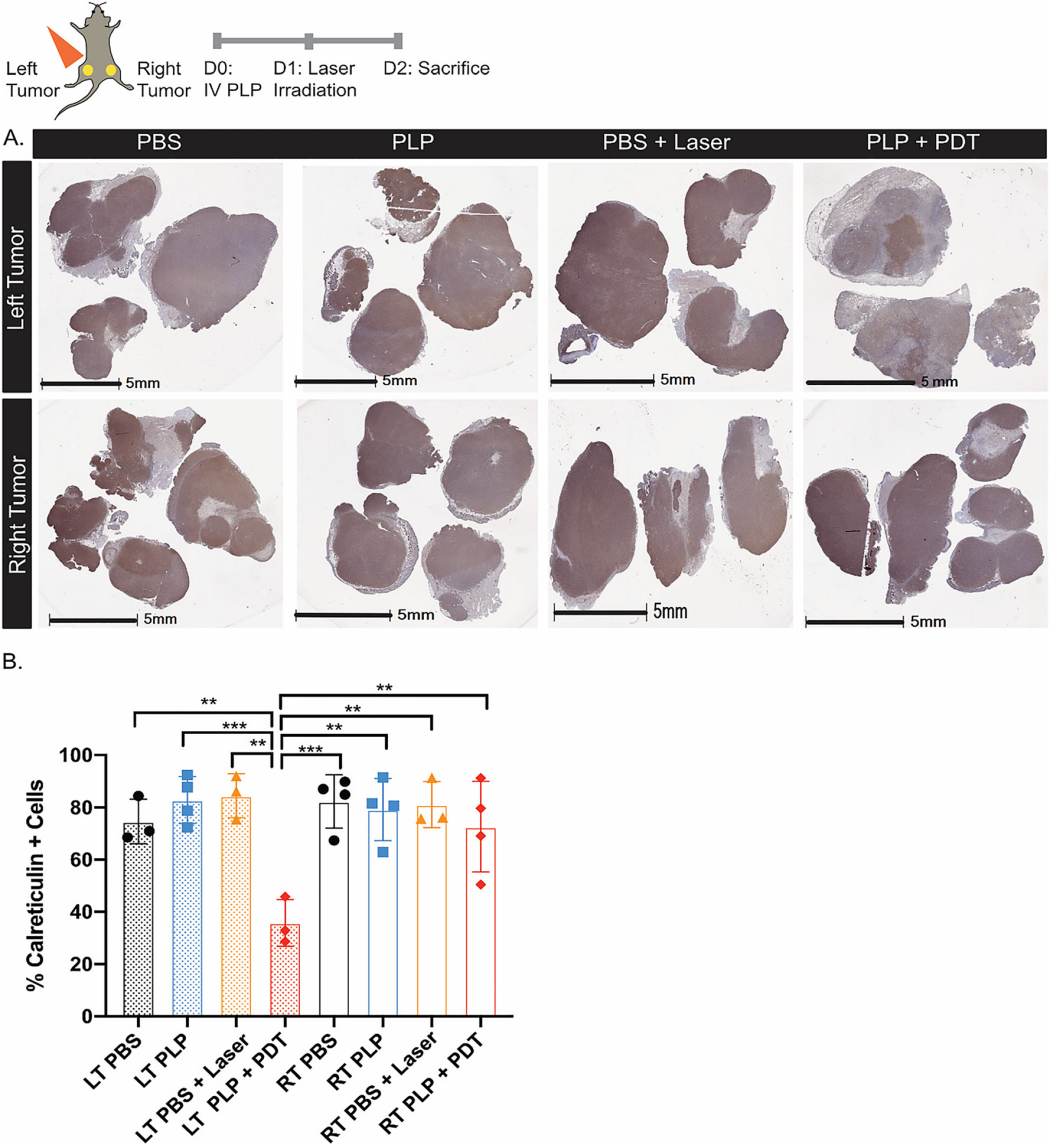
PDT reduced calreticulin expression in irradiated tumors. Dual AE17-OVA+ tumor bearing mice were treated once with either PBS, PLP, PBS + laser irradiation, or PLP + laser irradiation. Twenty-four hours after irradiation, irradiated and nonirradiated tumors were collected and processed for immunohistochemistry. (A) Whole slide scan of calreticulin stained tumors in mice for irradiated tumors on the left flank and nonirradiated tumors on the right flank. Scale bars represent 5 mm. (B) Quantification of the whole tissue slide by Halo (*n* = 3–4 per group) for the left (LT) and right (RT) tumors. Data are mean ± standard deviation. Statistical significance was determined using a one-way ANOVA followed by a post-hoc Tukey test. *p < 0.05, **p < 0.01, ***p < 0.001.

Photodynamic therapy can induce the expression of damage-associated molecular patterns, such as calreticulin and heat shock proteins, which trigger immunogenic cell death. Calreticulin is typically found in the lumen of the endoplasmic reticulum, but localizes to the plasma membrane in PDT-treated tumor cells, where it serves as an “eat-me” signal to antigen-presenting cells [20]. As a known mediator of immunogenic cell death, we investigated how PDT affected calreticulin levels in AE17-OVA+ tumors. Dual AE17-OVA+ tumor bearing mice were treated with either PBS, PLP, PBS + laser irradiation, or PLP + laser irradiation. Twenty-four hours after irradiation, mice were sacrificed and tumors were collected for assessment of calreticulin expression. Whole slide tumor images revealed that in tumors of control mice treated with PBS, PLP, or PBS + laser, calreticulin expression was high and uniformly expressed throughout the tumor (Figure 3A). In contrast, in the irradiated tumors on the left flank of mice treated with PLP + PDT, tumor expression of calreticulin was reduced and clustered in certain regions of the tumor. Quantification of calreticulin staining revealed a 2.0-fold decline in expression for the irradiated tumors on the left flank of mice treated with PLP + PDT, relative to the non-irradiated tumors on the right flank (Figure 3B). This reduction suggests a potential role for calreticulin in the immune response after PDT.

### 3.4 Four repeated cycles of photodynamic therapy elicited an abscopal effect

**Figure 4:**
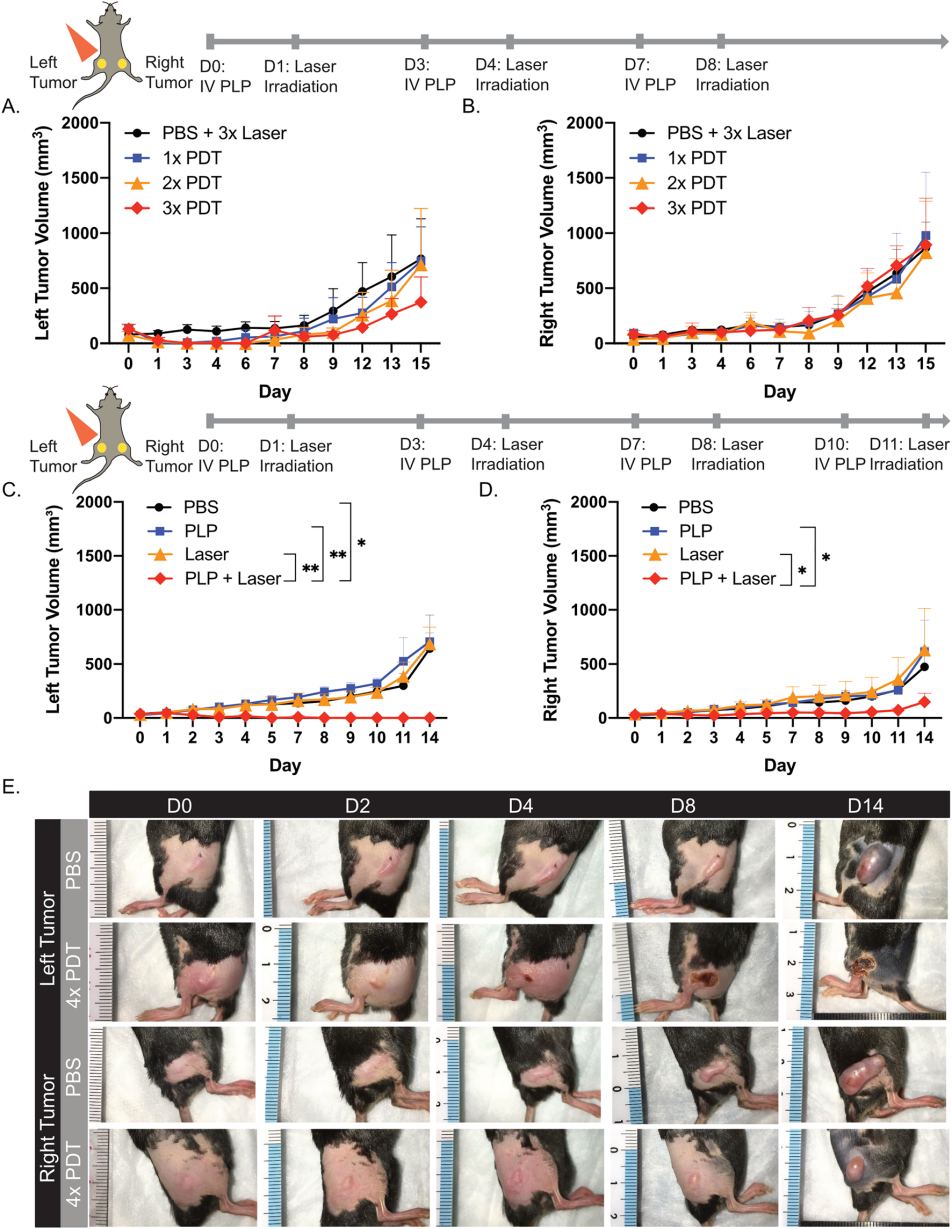
Four repeated cycles of PDT induced an abscopal effect. (A) Dual subcutaneous AE17-OVA+ tumor bearing mice were treated with either PBS and laser irradiation at 50 J/cm^2^ three times (*n* = 6), or PLP and laser irradiation at 50 J/cm^2^ one (*n* = 5), two (*n* = 6), or three times (*n* = 5). Evaluation of irradiated, left hindlimb tumor volumes. (B) Assessment of nonirradiated, right hindlimb tumor volumes. (C) Dual subcutaneous AE17-OVA+ tumor bearing mice were treated with four cycles of PBS, PLP (4 mg/kg), laser irradiation at 50 J/cm^2^, or PDT (PLP + laser). Tumors for irradiated, left tumor volumes were measured. (D) Nonirradiated, right hindlimb tumor volumes were measured (*n* = 4). (E) Photos of left, irradiated and right, nonirradiated hindlimb tumors of mice treated with either PBS or four cycles of PDT on days 0, 2, 4, 8, and 14. Experiments were repeated twice more with similar findings for left and right hindlimb tumor volumes. Data are mean ± standard deviation. Statistical significance was determined using a one-way ANOVA followed by a post hoc Tukey test. *p < 0.05, **p < 0.01.

As the immune system often requires priming to elicit sustained, long-term responses, the effect of repeated cycles of PDT on distal, nonirradiated tumor growth was evaluated. Mice with tumors treated with PDT once, twice, and three times experienced an initial regression at the irradiated site, but residual tumor cells induced tumor regrowth (Figure 4A). For nonirradiated, right hindlimb tumors, PDT performed up to three times did not delay AE17-OVA+ tumor growth, relative to mice receiving laser control treatments (Figure 4B).

However, when PDT was performed four times, irradiated tumors on the left hindlimb were eradicated (Figure 4C and E), while nonirradiated tumors on the right hindlimb experienced a delay in tumor growth (Figure 4C and E). Two weeks after the initial injection of PLP, mice that received PDT four times had 4.2-fold and 4.1-fold smaller tumors relative to laser and PLP controls, respectively. As such, repeated priming was necessary to mount an abscopal effect that could delay the growth of established, distal tumors.

### 3.5 CD4+ and CD8+ T cells participated in the abscopal effect

To investigate the immune response underlying the abscopal effect, dual subcutaneous AE17-OVA+ tumor bearing mice were treated with PDT four times. Subsequently, spleens and nonirradiated right hindlimb tumors were dissected for flow cytometry, and serum was collected for cytokine multiplexing.

**Figure 5:**
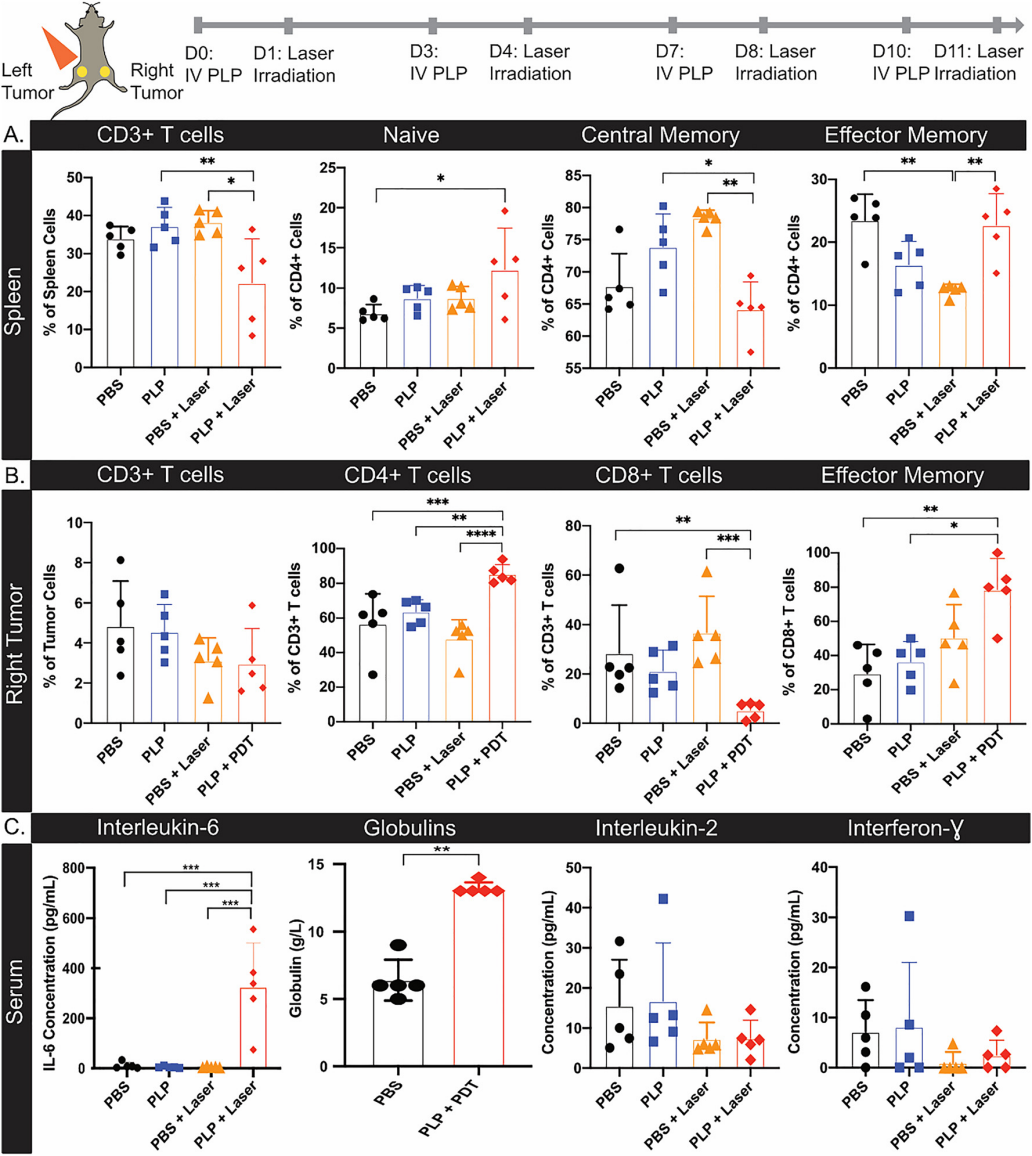
The abscopal effect involved CD4+ and CD8+ T cells. (A) Dual subcutaneous AE17-OVA+ tumor bearing mice were treated with four cycles of PBS, PLP (4 mg/kg), laser irradiation at 50 J/cm^2^, or PDT (PLP + laser). Mice were sacrificed on day 14 and spleens and right tumors were harvested for flow cytometry, while serum was collected for cytokine analysis. Summary of the percentage of CD3+ T cells, naive, central memory, and effector memory CD4+ T cells in the spleen of treated mice. (B) Summary of the percentage of CD3+ T cells, CD4+ T cells, CD8+ T cells, and effector memory CD8+ T cells in the non-irradiated, right tumors of treated mice. (C) Assessment of the concentration of interleukin-6, globulins, interleukin-2, and interferon-gamma in serum of mice (*n* = 5). Data are mean ± standard deviation. Statistical significance was determined using a one-way ANOVA, followed by a post hoc Tukey test. *n* = 5, *p < 0.05, **p < 0.01, ***p < 0.001, ****p < 0.0001.

In the spleen, there was a significantly smaller percentage of CD3+ T cells after PDT, relative to mice that received PLP or PBS + laser irradiation (Figure 5A). While the proportion of CD4+ and CD8+ T cells did not differ between the treatment groups (Supplementary Figure 4), differences were observed in the percentage of naive, central memory, and effector memory CD4+ T cells (Figure 5A) between PDT treated mice and controls. There was a 1.8-fold increase in naïve CD4+ T cells in the spleens of PDT treated mice, compared to PBS controls. Additionally, there was a decline in the percentage of central memory CD4+ T cells, compared to PLP and PBS + laser treated mice. Furthermore, there was a 1.8-fold increase in effector memory CD4+ T cells after PDT, relative to PBS + laser treated mice. In contrast, no differences in the proportions of naive, effector memory, and central memory CD8+ T cells were observed in the spleens of PDT treated mice, compared to controls (Supplementary Figure 4). As such, repeated PLP mediated-PDT may induce proliferation of naïve CD4+ T cells and localization of effector memory CD4+ T cells in the spleen, whereas central memory T cells may migrate from the spleen.

In the right, nonirradiated tumor, the percentage of CD3+ T cells was similar across all treatment groups. Upon examining T-cell subpopulations, we observed that there was an increase in the percentage of CD4+ T cells in the nonirradiated, right hindlimb tumor of mice that received PDT, relative to all treatment controls (Figure 5B). Notably, the percentage of CD8+ T cells was 5.4-fold and 7.0-fold lower in the nonirradiated tumor after PDT, compared to PBS and PBS + laser treated mice respectively. Moreover, the proportion of effector memory CD8+ T cells was 2.2-fold and 2.7-fold higher in the right, nonirradiated tumors, relative to PLP or PBS treated mice. No differences in central memory or naive CD8+ T cells were observed across the different treatment groups (Supplementary Figure 5). Taken together, the lower percentage of CD8+ T cells present in the nonirradiated tumor, in combination with the higher proportion of effector memory CD8+ T cells in PDT-treated mice, suggests reduced persistence and exhaustion of CD8+ T cells, as they mount a cytotoxic response to control distal, nonirradiated tumors.

To assess the systemic effects of repeated PDT, serum was also obtained for cytokine analysis. IL-6 levels in the serum were significantly increased relative to controls (30.8, 66.8, and 52.1-fold over PBS, PLP and laser controls), indicative of acute inflammation (Figure 5C). Meanwhile, serum globulins for PLP + PDT treated mice were 2.1-fold greater than in PBS treated mice (Figure 5C), suggestive of CD4+ T cells providing help to activate B cells and inducing the secretion of gamma globulins after repeated PDT. However, further delineation between alpha, beta, and gamma globulins are needed. Levels of other pro-inflammatory cytokines in the serum, including IL-2 and IFN-γ did not differ between the treatment groups (Figure 5C). This finding provides further evidence of T cell exhaustion, as these inflammatory cytokines have been reported to increase in serum after PDT [12, 13]. Similarly, the levels of other cytokines, including IL-1β, TNF-α, GM-CSF, IL-2, IL-4, and IL-12p70, and the chemokine, MCP-1, did not differ between the different treatment groups (Supplementary Figure 6). The levels of the anti-inflammatory cytokine, IL-10, in the serum were similar between the treatment groups.

A preliminary assessment on the effect of repeated PDT on liver function was conducted (Supplementary Figure 7). Hematoxylin and eosin staining of the liver collected from mice that received four cycles of PDT did not reveal signs of inflammation (Supplementary Figure 7A). Moreover, there were no significant differences in levels of serum alanine transferase, amylase, total bilirubin, calcium, phosphorus, and sodium (Supplementary Figure 7B) in mice that received four cycles of PDT relative to PBS-treated mice.

### 3.6 Combination PDT and αPD-1 antibody therapy improved survival compared to monotherapy

**Figure 6:**
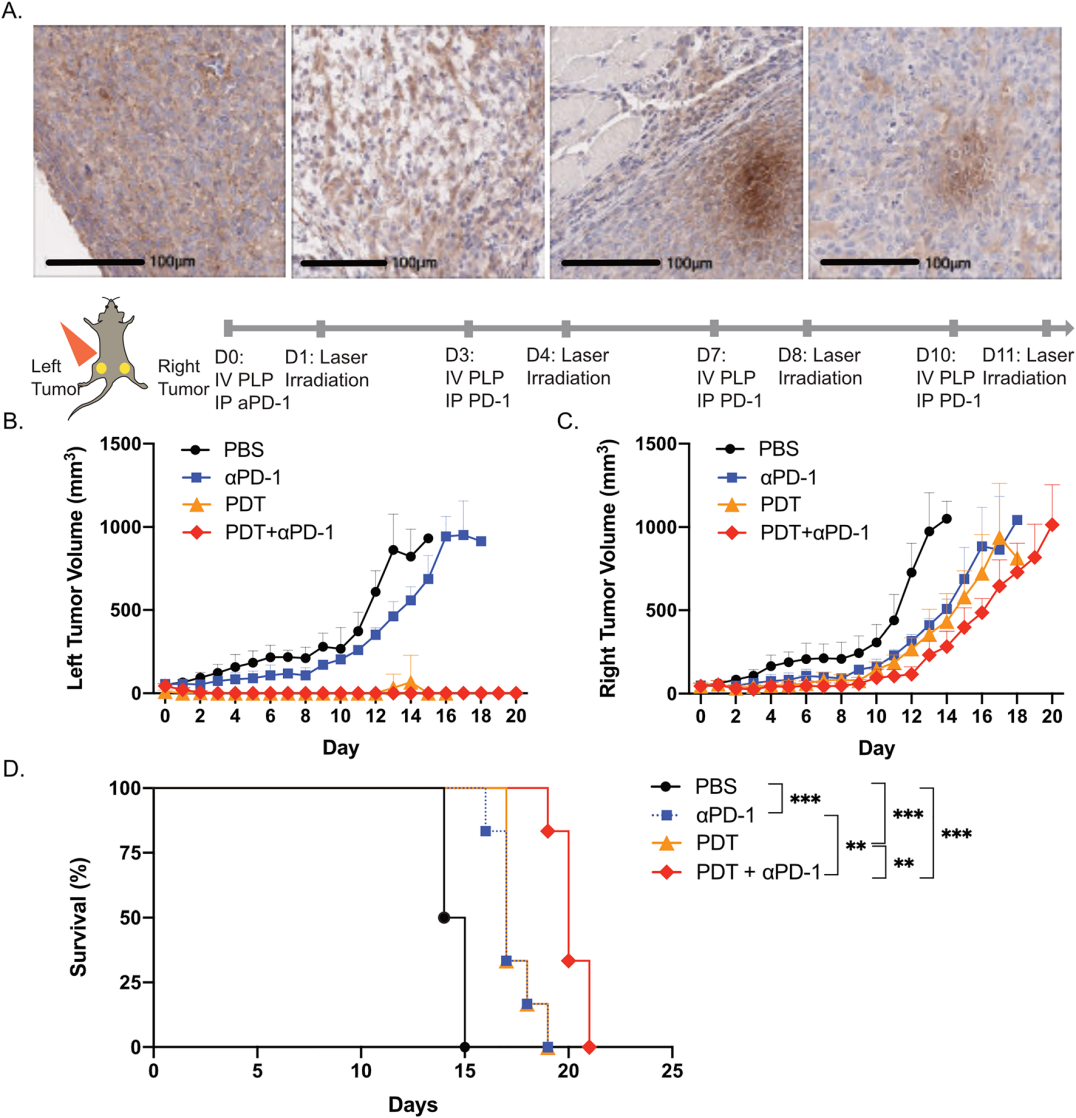
Combination therapy with PDT and αPD-1 antibody treatment improved survival relative to monotherapy. (A) Microscopic images of PD-1 stained AE17-OVA+ tumors. Dual subcutaneous AE17-OVA+ tumor bearing mice were treated with four cycles of PBS, αPD-1 antibody (12 mg/kg), PDT (PLP at 4 mg/kg), or combination αPD-1 antibody (12 mg/kg) and PDT (PLP at 4 mg/kg). Scale bars represent 100 μm. (B) Assessment of irradiated, left hindlimb tumors of mice receiving the aforementioned treatments. (C) Assessment of nonirradiated, right hindlimb tumors of mice receiving the aforementioned treatments. (D) Survival of mice receiving the aforementioned treatments. Data are mean ± standard deviation. Statistical significance of the survival of mice was determined using a one-way ANOVA, followed by a post hoc Tukey test (*n* = 5, **p < 0.01, ***p < 0.001).

With the low persistence of CD8+ T cells in nonirradiated tumors and the lack of increase in serum pro-inflammatory cytokines, including IL-2, IFN-γ, and TNF-α, we posited that repeated PDT induced T-cell exhaustion. Therefore, we sought to evaluate the role of immune checkpoint inhibitor treatment with αPD-1 antibody to complement PDT as a combination therapy in our AE17-OVA+ mouse mesothelioma model. Preliminary data from the double blind, randomized phase III CONFIRM clinical trial showed that single agent nivolumab, an αPD-1 antibody, improved overall survival in patients with malignant mesothelioma (median: 9.2 vs. 6.6 months) [21]. AE17-OVA+ tumors were strongly positive for PD-1 expression on immunohistochemistry (Figure 6A). Accordingly, we explored the antitumor effects of combination PDT and αPD-1 antibody therapy.

Dual subcutaneous AE17-OVA+ tumor bearing C57BL/6 mice were injected intravenously with PLP and intraperitoneally with 12.5 mg/kg of αPD-1 antibody four times over the course of 10 days. Twenty-four hours after PLP injection, the left hindlimb tumors were irradiated. In mice that received PDT or combination PDT + αPD-1 antibody, irradiated tumors on the left hindlimb were eradicated, whereas mice that received αPD-1 antibody treatment alone experienced steady tumor growth (Figure 6B). Meanwhile, for nonirradiated tumors on the right hindlimb, mice treated with combination therapy had the greatest delay in tumor growth (Figure 6C), which facilitated a significant survival advantage relative to monotherapy PDT or αPD-1 antibody treated mice. Meanwhile, monotherapy-treated mice had prolonged survival relative to PBS-treated mice.

## 4 Discussion

In this work, we showed that PLP is a photosensitizer that can induce the abscopal effect, without having to combine chemotherapeutics or immunotherapies, in a highly aggressive, established tumor model. To further potentiate the immune response, PDT in combination with αPD-1 antibody improved antitumor efficacy. We previously reported that PLP can induce apoptosis in tumor cells after PDT in H460 lung tumors in mice [22], in VX2 buccal carcinomas in rabbits [15], and 9Lluc gliomas in mice [14]. Meanwhile, porphyrin-lipid, as a photosensitizer, incorporated into zinc nanoparticles and polymeric core–shell nanoparticles, has been reported to induce apoptosis and necrosis, triggering upregulation of calreticulin expression in 4T1 breast and CT26 colorectal tumor cells, respectively [12, 13]. Calreticulin serves as an “eat-me” signal to phagocytes and is a marker of ICD. The timepoint at which calreticulin is examined as well as the tumor model investigated can affect calreticulin expression. Previously, Duan et al. showed an increase in calreticulin in an *in vivo* 4T1 tumor model in Balb/c mice three days after PDT with pyrolipid as a photosensitizer [12]. Meanwhile, Korbelik at al. also reported an increase in calreticulin expression 1 h post-PDT in subcutaneous LLC tumors in C57Bl/6 mice [23]. In contrast, we report a decline in the expression of calreticulin in the irradiated tumors 24 h after PDT. This decline may reflect various possibilities, including: (1) Tumor cells may downregulate calreticulin in the lumen of the endoplasmic reticulum, (2) dying cells that have expressed calreticulin on the surface may have been cleared by the immune system after 24 h, resulting in a lower percentage of positive cells. The role of calreticulin in ICD in our AE17-OVA+ model necessitates additional investigation.

Four cycles of PDT were necessary to stimulate an immune system sufficient to delay distal tumor growth. This finding was similar to He et al., who used porphyrin-lipid as a photosensitizer in a nanoscale coordination polymer core–shell nanoparticle. They observed that three cycles of PDT with their porphyrin-lipid core–shell nanoparticle could delay distant tumor growth [13]. Underpinning this immune response, in the spleen, we observed a greater percentage of naïve and effector memory CD4+ T cells after four cycles of PDT relative to controls, and a decline in the percentage of central memory CD4+ T cells relative to controls. The reduction in central memory CD4+ T cells may be attributed to their reactivation, differentiation, and migration to the tumor [24]. This is supported by our observation of an increase in CD4+ T cells in the nonirradiated tumor. We speculate that CD4+ T cells are providing help to CD8+ T cells, which can support CD8+ effector memory formation and maintenance [25]. Helped CD8+ T cells then upregulate molecules such as granzyme A, granzyme B, perforin, fas ligand, trail, and IFNγ, which play roles in cytotoxic effector function, and may help delay distal tumor growth. Moreover, we observed that serum globulins doubled in mice that received four cycles of PDT relative to mice treated with PBS. This finding suggests that there may be an increase in gamma globulins (i.e. antibodies), which could arise from CD4+ T cells activating B cells to induce secretion of antibodies [26]. A single round of PDT using Tookad (WST11) has previously been reported to increase serum IgG titers in CT26 colorectal-tumor bearing mice [27]. Further investigation is needed to determine the role of humoral immunity in inducing the abscopal effect after repeated PDT.

**Figure 7:**
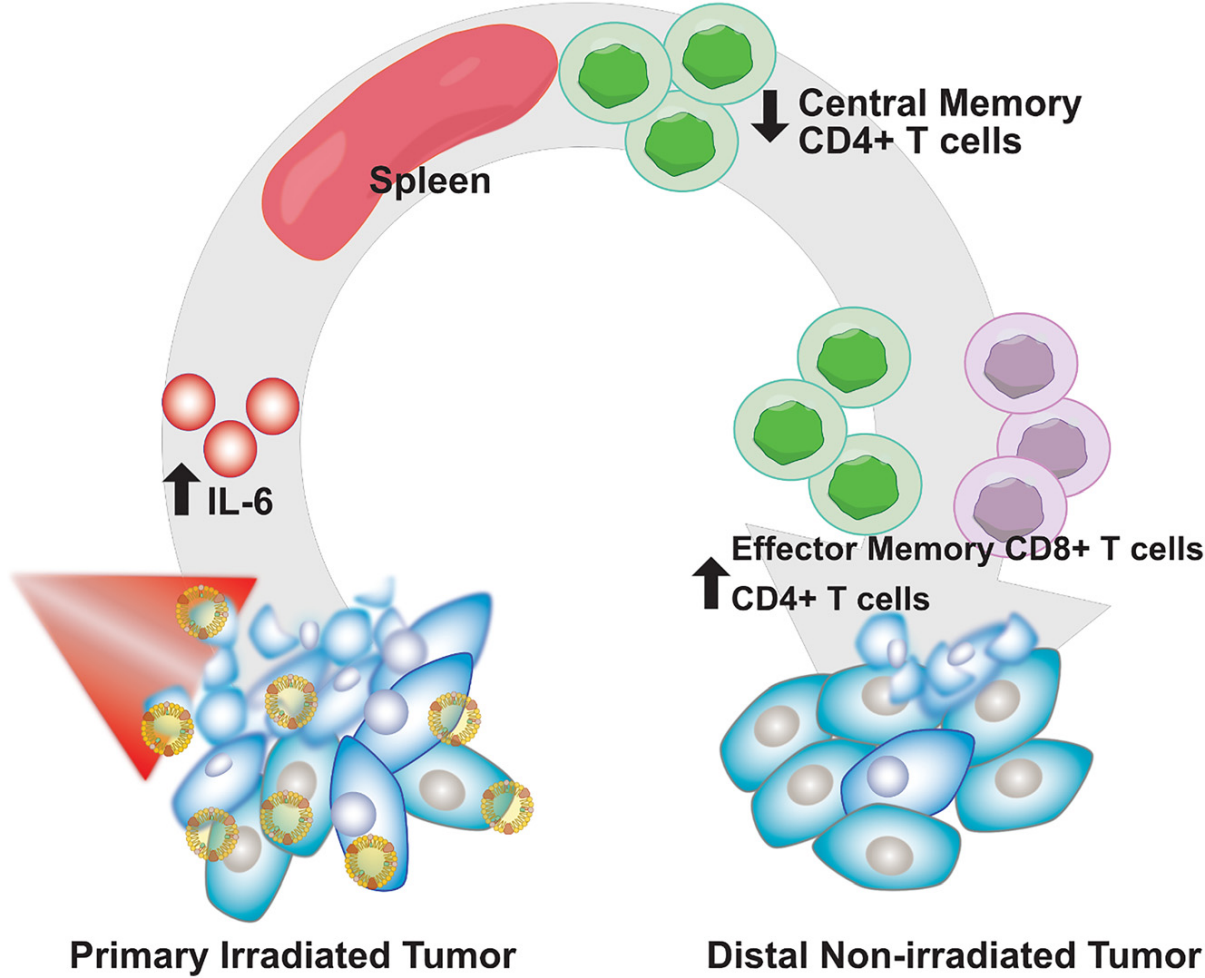
Schematic of immune mechanism underlying the abscopal effect induced by PLP-mediated PDT.

In the nonirradiated, right hindlimb tumors, we observed a reduction in the percentage of CD8+ T cells after four cycles of PDT, relative to control mice. Of the CD8+ T cells present in the tumor, a greater percentage had an effector memory phenotype compared to control mice. CD8+ T cells are critical to antitumor effects. For instance, CD8+ T cells are necessary to inhibit distant lung metastases in a murine EMT6 breast tumor model after PDT with Photofrin [28]. Similarly, Mroz et al. reported an increase in CD8+ T-cell infiltration in distal CT26.CL25 colorectal tumors after treatment of a primary tumor with Verteporfin-mediated PDT [29]. In our study, the presence of effector memory CD8+ T cells in the tumor is likely a product of the initial activation of innate immunity and downstream triggering of adaptive immunity. The increase in serum IL-6 after four cycles of PLP-mediated PDT implies that the innate immune system was activated, and likely facilitated the recruitment of neutrophils, dendritic cells, and macrophages [30]. In turn, dendritic cells prime naïve CD8+ T cells, resulting in differentiation into effector CD8+ T cells, and further differentiation into the effector memory phenotype. We posit that these CD8+ effector memory T cells migrated to the distal tumor and induced cytotoxic effects, helping to delay distal tumor growth (Figure 7). However, due to the aggressiveness of AE17-OVA+ tumors, antitumor immunity was overwhelmed – CD8+ T cells became exhausted, proliferative capacity, and persistence declined, accounting for the reduction in CD8+ T cells in the tumor.

Our conjecture of T-cell exhaustion is further supported by our serum cytokine analysis. We did not observe any increases in the levels of pro-inflammatory cytokines, such as IL-2 and IFNγ, which contrasts with many reports on photodynamic therapy. In CT26 colorectal tumor-bearing mice, a single round of PDT with porphyrin-lipid photosensitizer incorporated into a polymeric core–shell nanoparticle increased serum TNF-α, IFNγ, and IL-2 the day after PDT [13]. Meanwhile, zinc nanoparticles loaded with porphyrin-lipid photosensitizer used for PDT in 4T1 breast tumors also reported an increase in serum TNF-α, IFNγ, and IL-2, one day after PDT [12]. Our findings are consistent with characteristics of T-cell exhaustion, in which persistent antigen exposure can lead to loss of effector CD4+ and CD8+ T-cell function, thereby resulting in reduced proliferative capacity and production of pro-inflammatory cytokines such as IL-2, IFNγ, and TNF-α [31].

To further augment the antitumor efficacy of PDT and to improve control of both local and distant disease, we evaluated combination PDT with PD-1 antibody treatment. Together, both therapies potentiated antitumor effects and prolonged survival of AE17-OVA+ tumor bearing mice, relative to single therapy. These observations are consistent with others who have reported improved tumor growth control with combination PDT and PD-1 antibody treatment [12, 13, 32]. Immune checkpoint blockade has been evaluated in mesothelioma patients. Nivolumab in combination with ipilimumab can extend survival of patients with unresectable malignant pleural mesothelioma compared to chemotherapy (median: 18.5 vs. 14.1 months) [33]. These encouraging findings prompted the Food and Drug Administration (FDA) to approve combination nivolumab and ipilimumab for first line treatment for patients with unresectable malignant pleural mesothelioma. In addition to PD-1 and CTLA-4, other checkpoint targets (i.e. LAG-3, OX-40, and CD137) are interesting combination therapy candidates. Moreover, as PLP is a theranostic nanoparticle that can deliver a payload, it is of interest to investigate its ability to deliver adjuvants and other immunostimulatory molecules (i.e. STING agonists) that further potentiate antitumor immunity. In conjunction with immune stimulation, strategies that target the tumor microenvironment to improve the antitumor efficacy of PDT is of growing interest [34]. One notable strategy involved manganese dioxide nanoparticles loaded with doxorubicin and chlorin e6, which induced the decomposition of tumor H_2_O_2_ to alleviate tumor hypoxia and enhance antitumor efficacy [35]. Future work will center on improving the tumor milieu in which PDT can synergize with immunotherapies.

## 5 Conclusions

PLP-mediated PDT can induce the abscopal effect in highly aggressive tumors. This immune response was further potentiated in combination with αPD-1 antibody treatment and improved survival of mice with AE17-OVA+ mesotheliomas relative to PDT or αPD-1 antibody treatment alone. Accordingly, PLP-mediated PDT in combination therapy shows promise in generating a systemic immune response to improve outcomes for patients with metastatic cancers.

## Supplementary Material

Supplementary Material
