# Supplementary material for: Repeated porphyrin lipoprotein-based photodynamic therapy controls distant disease in mouse mesothelioma via the abscopal effect
Source: Nanophotonics. 2021 Aug 3;10(12):3279–94. doi: 10.1515/nanoph-2021-0241 (PMC9646247; doi:10.1515/nanoph-2021-0241)
Supplement: Supplementary file 1 — Supplementary Material [file j_nanoph-2021-0241_suppl.docx]

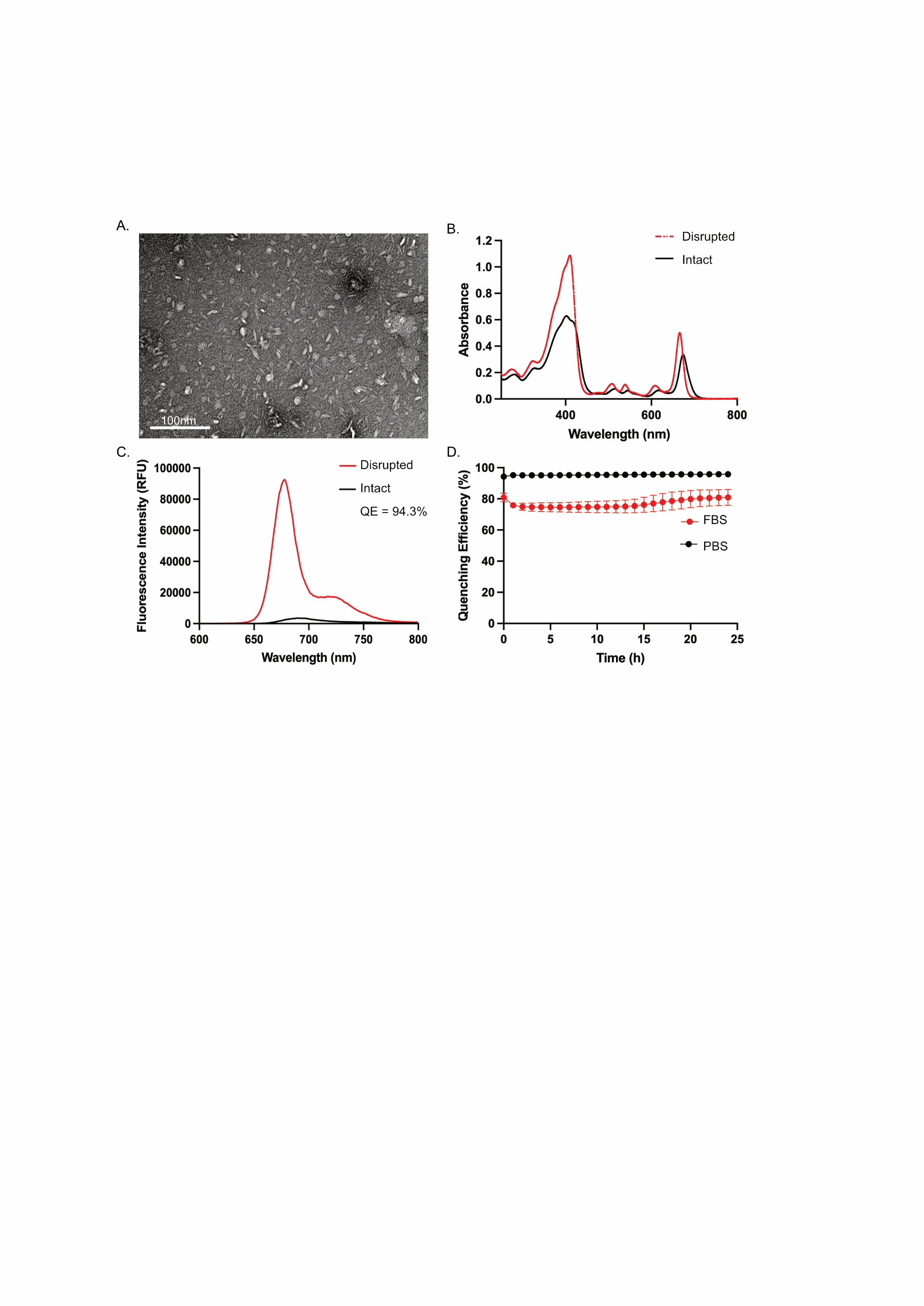


Supplementary Figure 1. Characterization of PLP. A) Transmission electron microscopy image of PLP after 30 s staining with 1% uranyl acetate。Samples were imaged under 200 000x magnification. B) Ultraviolet-visible spectroscopy was used to assess the absorbance of PLP in PBS and upon disruption in methanol. C) Fluorescence spectra of PLP upon excitation at 410 nm. Emission was collected between 500 to 799 nm. Samples were measured in PBS and disrupted in 1% TritonX-100. D) PLP serum stability was determined using the quenching efficiency of PLP in PBS (intact) and 1% TritonX-100 (disrupted) over the course of 24 h. PLP was excited at 410 nm and emission was collected from 500 to 799 nm (n=3).


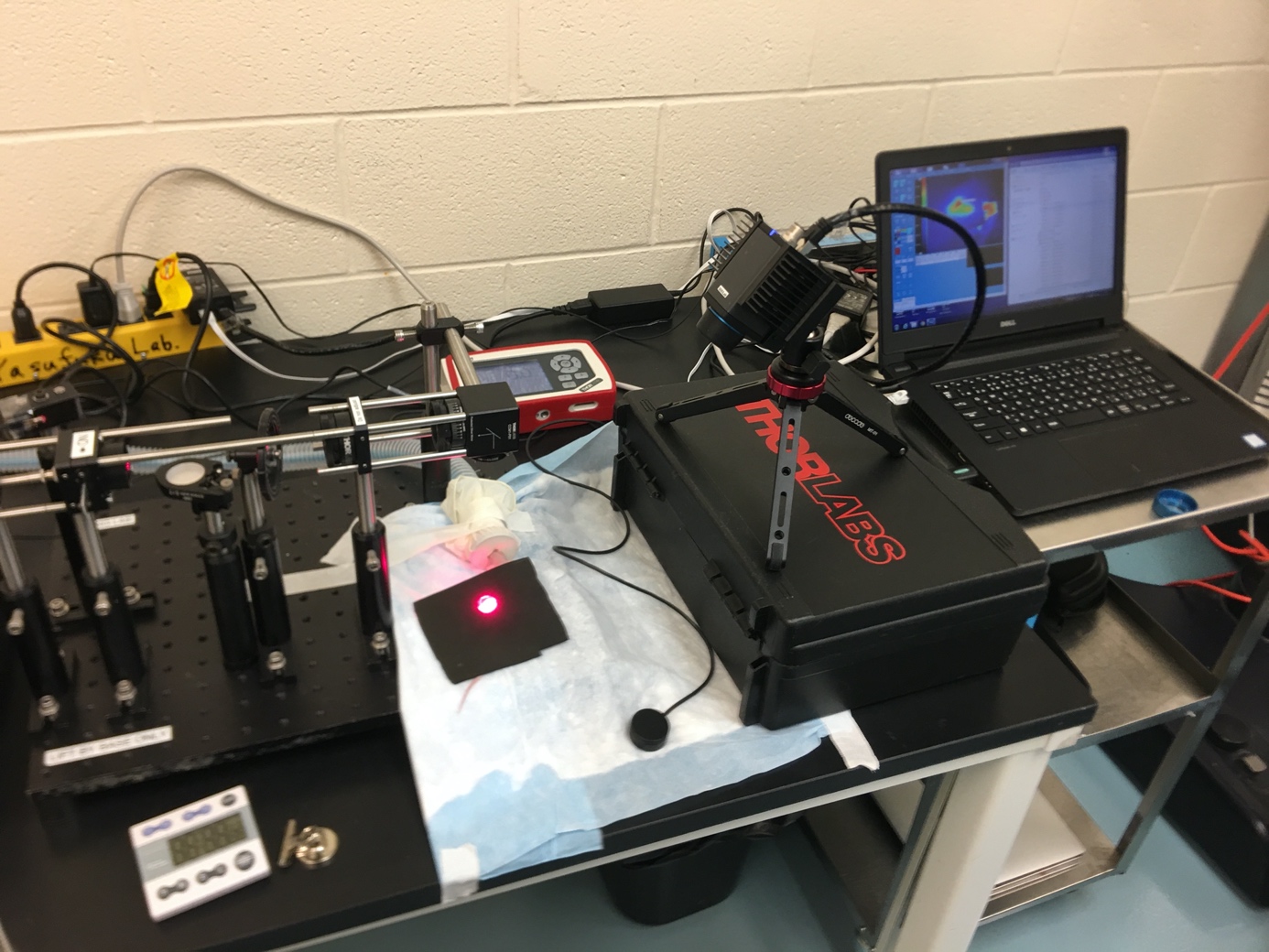
Supplementary Figure 2. Photograph of custom laser set-up for PDT.


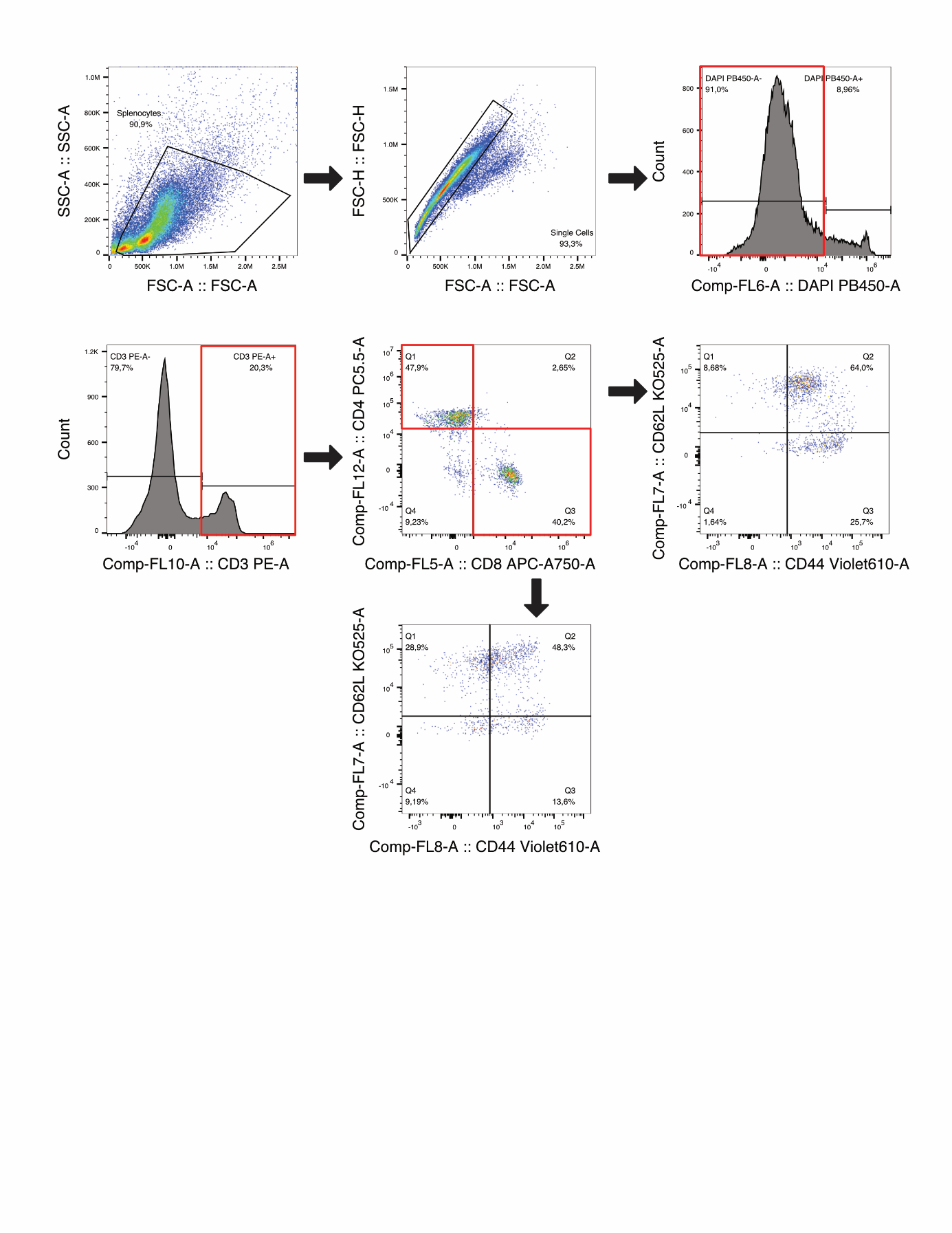


Supplementary Figure 3. Gating strategy for effector and central memory and naïve CD4+ and CD8+ T cells. The above plot is representative of our approach for both the spleen and tumor.


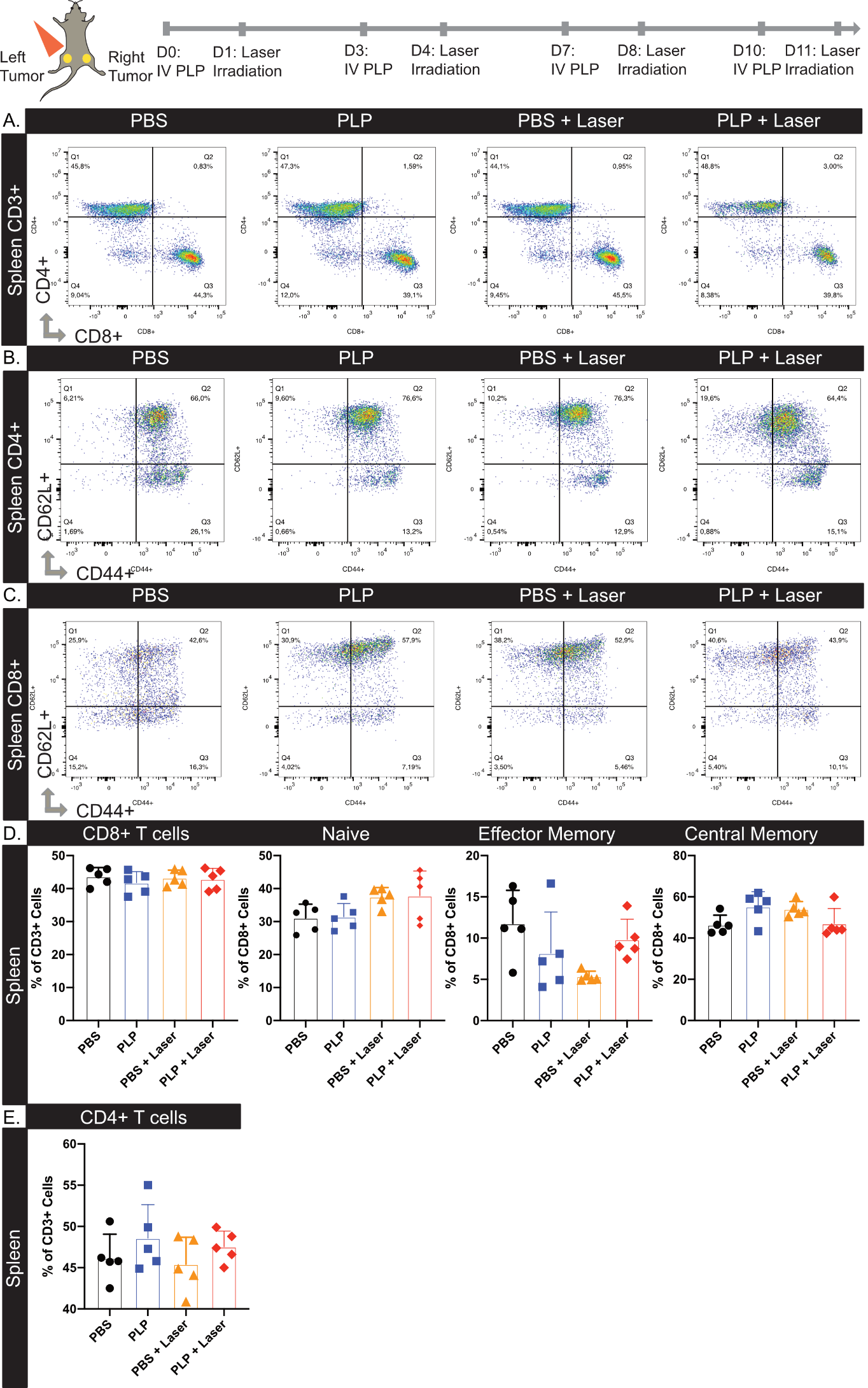


Supplementary Figure 4. T cell sub-populations in the spleen after four cycles of PDT. Dual subcutaneous AE17-OVA+ tumor bearing mice were treated with four cycles of PBS, PLP (4 mg/kg), laser irradiation at 50 J/cm^2^, or PDT (PLP + Laser). Mice were sacrificed on day 14 and spleens were harvested for flow cytometry. Representative flow cytometry plots of: A) CD4+ and CD8+ T cells, B) CD4+ naïve (CD62L+CD44-), central memory (CD62L+CD44+), effector memory (CD62L-CD44+), C) CD8+ naïve (CD62L+, CD44-), central memory (CD62L+CD44+), effector memory (CD62L-CD44+) T cells from spleens of mice that received the aforementioned treatments. Summary of: D) CD8+ T cells and sub-populations of naïve, effector memory, and central memory CD8+ T cells, and E) CD4+ T cells in the spleens of mice that received the four aforementioned treatments (n=5 per group). Data are mean ± standard deviation. Statistical significance was determined using a one-way ANOVA, followed by a post-hoc Tukey test.


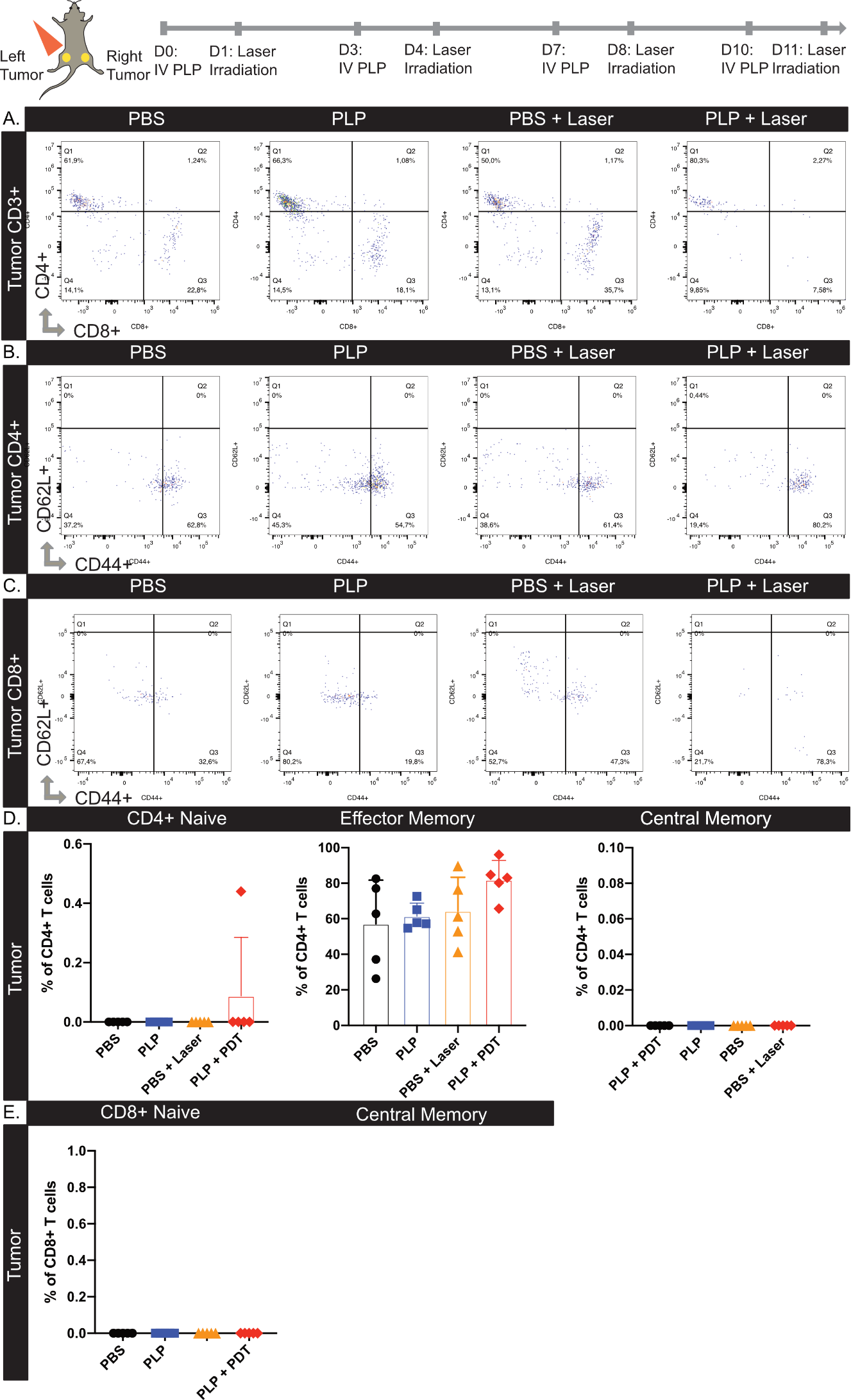


Supplementary Figure 5. T cells in the non-irradiated, right hindlimb tumors after 4 cycles of PDT. Dual subcutaneous AE17-OVA+ tumor bearing mice were treated with four cycles of PBS, PLP (4 mg/kg), laser irradiation at 50 J/cm^2^, or PDT (PLP + Laser). Mice were sacrificed on day 14 and tumors were harvested for flow cytometry. Representative flow cytometry plots of: A) CD4+ and CD8+ T cells, B) CD4+ naïve (CD62L+CD44-), central memory (CD62L+CD44+), effector memory (CD62L-CD44+), C) CD8+ naïve (CD62L+, CD44-), central memory (CD62L+CD44+), effector memory (CD62L-CD44+) T cells from non-irradiated tumors of mice that received the aforementioned treatments. Summary of: D) CD8+ T cells and sub-populations of naïve, effector memory, and central memory CD8+ T cells and E) CD4+ T cells in the non-irradiated tumors of mice that received the four aforementioned treatments (n=5 per group). Data are mean ± standard deviation. Statistical significance was determined using a one-way ANOVA, followed by a post-hoc Tukey test.


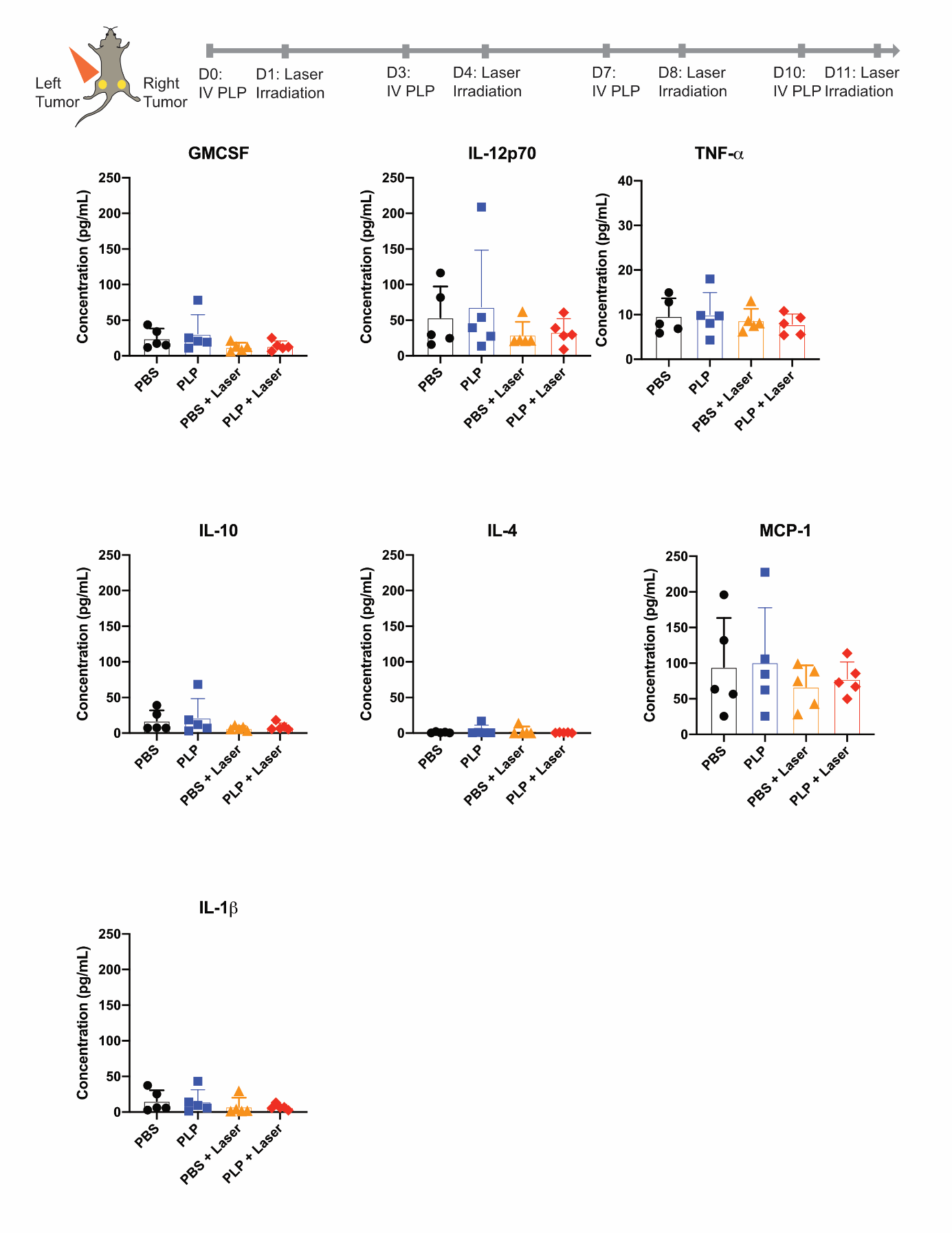


Supplementary Figure 6. Levels of various cytokines in the serum after repeated PDT. Dual subcutaneous AE17-OVA+ tumor bearing mice were treated with four cycles of PBS, PLP (4 mg/kg), laser irradiation at 50 J/cm^2^, or PDT (PLP + Laser). Mice were sacrificed on day 14 and serum was collected for analysis of various cytokines, including GM-CSF, IL-1$\beta$, IL-4, IL-10, IL-12p70, TNF-$\propto$, and MCP-1. Data are mean ± standard deviation. Statistical significance was determined using a one-way ANOVA, followed by a post-hoc Tukey test. (n=5 per group)


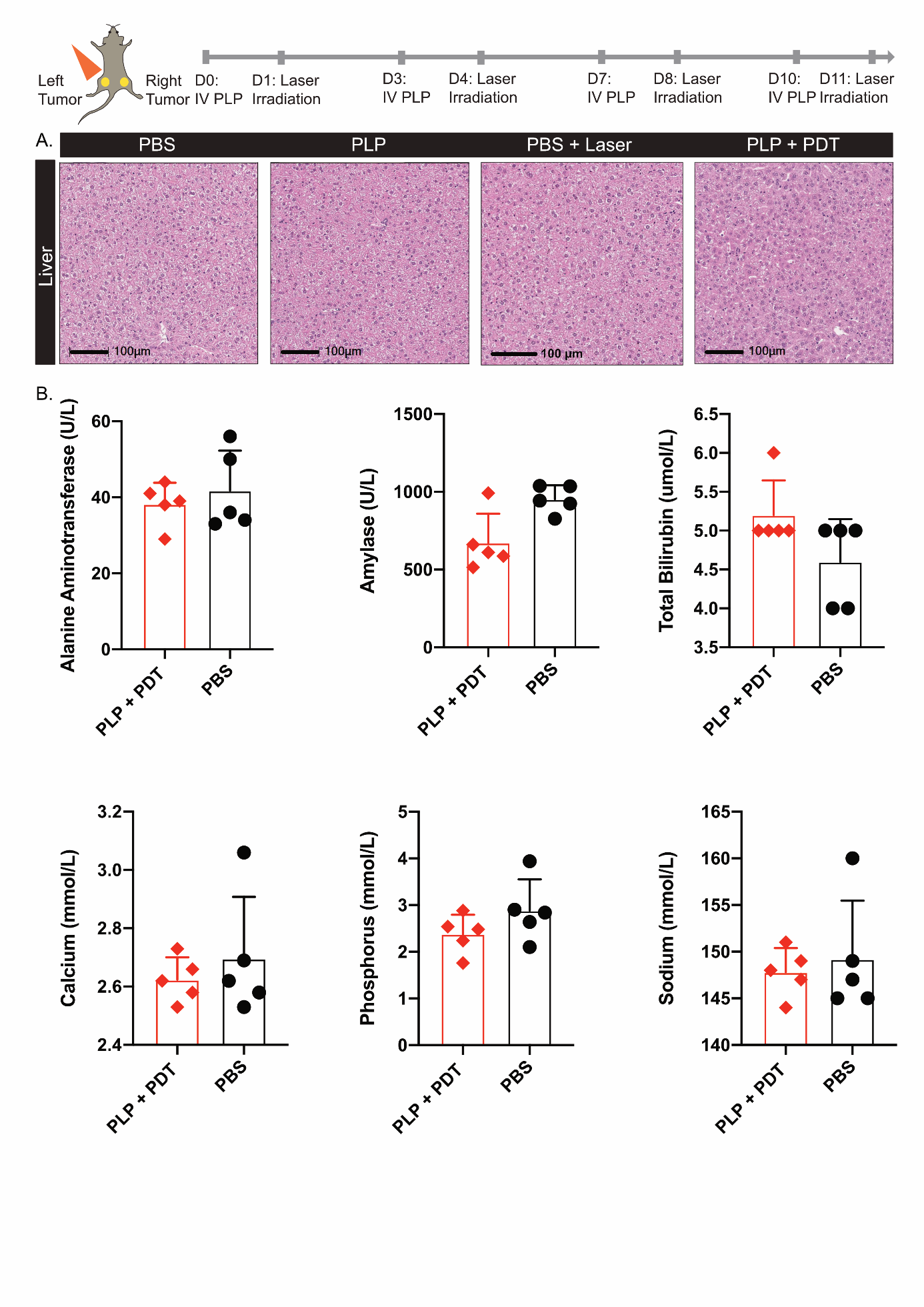


Supplementary Figure 7. Preliminary evidence of the safety of repeated PDT. Dual subcutaneous AE17-OVA+ tumor bearing mice were treated with four cycles of PBS, PLP (4 mg/kg), laser irradiation at 50 J/cm^2^, or PDT (PLP + Laser). Mice were sacrificed on day 14 and serum was collected for biochemistry analysis. A) Hematoxylin and eosin staining of liver tissue after treatment. B) Serum levels of alanine transferase, amylase, total bilirubin, calcium, phosphorus, and sodium in mice treated with four cycles of either PLP + PDT or PBS (n=5 per group). Data are mean ± standard deviation. Statistical significance was determined using an unpaired t-test and p<0.05.
